# Testicular Lmcd1 regulates phagocytosis by Sertoli cells through modulation of NFAT1/Txlna signaling pathway

**DOI:** 10.1111/acel.13217

**Published:** 2020-08-09

**Authors:** Xiaohang Jin, Sheng Zhang, Tianbing Ding, Pengtao Zhao, Chunli Zhang, Yuxing Zhang, Wei Li

**Affiliations:** ^1^ Department of Basic Medical Morphology Medical College Xijing University Xi'an China; ^2^ Department of Basic Functioning Medicine Medical College Xijing University Xi'an China; ^3^ Department of Human Anatomy, Histology and Embryology Fourth Military Medical University Xi'an China

**Keywords:** dephosphorylation, LMCD1, NFAT1, phagocytosis, Sertoli cells (SCs)

## Abstract

Increased oxidative stress is well known to cause testicular dysfunction in aging males, but the detailed relationships between aging, oxidative stress, and testicular function remain to be elucidated. LIM and cysteine‐rich domains 1 (LMCD1) regulates fundamentally cellular process by interacting with transcription factors. A recent study has identified *Lmcd1* as one of the most upregulated nuclear proteins associated with Sertoli cell (SC) differentiation, raising the possibility that testicular actions of LMCD1 are likely to take place. Herein, we reported that LMCD1 was exclusively expressed in the nuclei of SCs. This expression was regulated by TNF‐α signaling produced by apoptotic germ cells (GCs) and was suppressed by oxidative stress in a STAT3‐dependent manner. Ablation of endogenous LMCD1 expression caused lipid accumulation and senescence in GC co‐incubated SCs. Using a previously validated *in vivo* siRNA approach, we showed that LMCD1 depletion significantly impaired male fertility by inducing oligozoospermia and asthenospermia. Mechanistically, LMCD1 upregulation was associated with the nuclear enrichment of the nuclear factor of activated T cells 1 (NFAT1), a core component of Ca^2+^/calmodulin‐dependent pathway. LMCD1 facilitated the dephosphorylation and nuclear translocation of NFAT1, which consequently expedited the transactivation of *Txlna*, a binding partner of the syntaxin family essential for testicular phagocytosis, and thus promoted the removal of apoptotic GCs by phagocytic SCs. Collectively, LMCD1 may operate as a novel pretranscriptional integrator linking SC phagocytosis, lipid homeostasis, and cell senescence.

## Graphical abstract

SCs‐specific LMCD1 expression is compromised by oxidative stress in aged testis. LMCD1 regulates phagocytosis by Sertoli cells through modulation of NFAT1/Txlna signaling pathway.

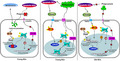

## 1 INTRODUCTION

During the complicated spermatogenesis, most germ cells (GCs) undergo apoptosis, followed by effective removal from germinal epithelium by Sertoli cells (SCs). This is so‐called testicular phagocytosis, which is essential for the maintenance of the inner environment homeostasis within seminiferous tubules. On the other hand, apoptotic spermatogenic cells are degraded to form lipids that are used to produce ATP, thus serving as potent energy sources for SCs (Xiong, Wang, Wu, Chen, & Han, 2009). So, phagocytosis is crucial for both GCs and SCs. Although the physiological responses of testicular phagocytosis have been well‐documented, the molecular basis underpinning this unique process is still in its infancy. We previously showed that α‐taxilin protein (TXLNA), a binding partner of the syntaxin family that functions as a central player in the intracellular vesicle traffic, functions as a critical regulator of testicular phagocytosis in SCs (Dong et al., 2016). Nevertheless, the transcriptional or post‐translational mechanisms responsible for controlling TXLNA expression are poorly understood.

Acting as essential components of the regulatory machinery in cells, LIM domain proteins are key factors modulating cell growth, differentiation, and cell fate determination under pathophysiological circumstances (Bian et al., 2010). LIM and cysteine‐rich domains 1 (LMCD1) was originally identified by screening of the expressed sequence tag database (dbEST). Northern blot analysis shows that *Lmcd1* is highly expressed in cardiac and skeletal muscles. Functionally, LMCD1 regulates cardiac hypertrophy (Bian et al., 2010) and thrombin‐induced smooth muscle cell proliferation (Janjanam et al., 2018) by interacting with different transcription factors. However, the physiological relevance of LMCD1 signaling beyond circulatory and musculoskeletal systems remains largely unknown.

Very recently, by using high‐throughput screening in rat SCs, Ryser S *et al*. have identified *Lmcd1* as one of the most upregulated nuclear proteins associated with SC differentiation (Ryser et al., 2011). Given that the expression of LIM domain proteins is emerging as a unique signature of SCs (Yang et al., 1998), it is tempting to propose that additional relevant roles of LMCD1 in the control of protein homeostasis at transcriptional level may also be provided with specific gonadal actions.

On the aforementioned basis, we aimed at assessing the expression pattern and biological relevance of LMCD1 signaling in testis. As our initial evidence demonstrated a steady upregulation of LMCD1 expression in SCs treated with apoptotic GCs, multiple experimental settings were used to define the paracrine regulation of this novel nuclear protein, as well as the molecular basis of its direct control of SC phagocytosis.

## 2 RESULTS

### 2.1 SC‐specific expression of LMCD1 in mouse testis

**FIGURE 1:**
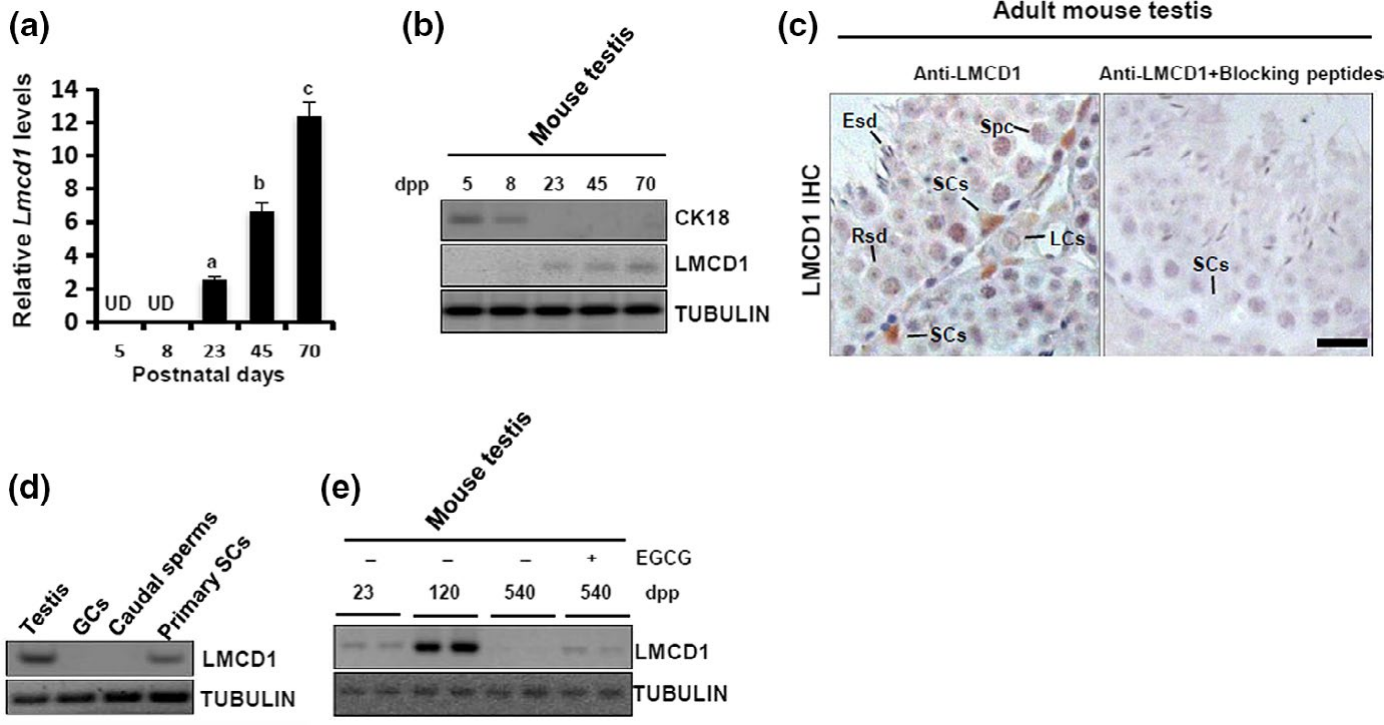
Testicular expression of LMCD1 is restricted to the nuclei of differentiated Sertoli cells (SCs), and this expression is compromised in aged testis. (a) *Lmcd1* mRNA level along the testicular development was assessed using RT‐qPCR. Amplification of *Gapdh* mRNA was used as an internal control. Quantitative values were presented as mean ± *SD* of three independent experiments. Different superscript letters denote groups that are statistically different (*p* < 0.05). UD, undetectable. (b) Immunoblotting analysis of LMCD1 expression along the postnatal testicular development. (c) Localization of LMCD1 protein in testicular sections from adult mouse was evaluated using immunohistochemistry. Scale bar, 20 μm. LCs, Leydig cells; Spc, primary spermatocyte; Rsd, round spermatid; Esd, elongated spermatid. (d) Expression profile of LMCD1 was evaluated in different spermatogenic cells and in mouse testis using immunoblotting. Tubulin served as a loading control. (e) 540‐day‐old mice received i.p. injections with EGCG as described in detail in EXPERIMENTAL PROCEDURES. Following EGCG treatment, testes from different ages of mice were subjected to immunoblotting analysis

Initial RT‐qPCR analysis was performed using testicular tissues from 5‐, 8‐, 23‐, 45‐, and 70‐day‐old mice (*n* = 5 per group), corresponding to the initiation of spermatogenesis, appearance of spermatogonia, completion of SC differentiation, appearance of adult‐staged adult Leydig cells (ALCs), and adult stages of postnatal maturation (Dong et al., 2016; Wu, Arumugam, Zhang, & Lee, 2010). *Lmcd1* expression began to emerge after SC differentiation (≧23‐day) and increased thereafter, with peak expression in adult testis (70‐day, Figure 1a). Immunoblotting analysis validated a precisely controlled appearance of LMCD1 in functionally mature SCs (as evidenced by the disappearance of CK18, the marker of immature SCs, at postnatal 23 days (Dong et al., 2016), at the translational level (Figure 1b and Figure S1). Consistently, immunohistochemistry showed the predominant presence of LMCD1 in the SCs (Figure 1c). The exclusive expression of LMCD1 in SCs was further verified in purified testicular cells (Figure 1d). Using immunoblotting, we observed that LMCD1 expression was totally abolished in 540‐day‐old testes, compared with a strong and a moderate expression in 120‐day‐old and 23‐day‐old testes, respectively. Intriguingly, i.p. injection with epigallocatechin‐3‐gallate (EGCG), a bioactive polyphenol from green tea that exerts a strong antioxidant activity (Ding et al., 2015), ameliorated age‐related oxidative stress (Figure S2) and partially restored the protein levels of LMCD1 in 540‐day‐old testes (Figure 1e).

### 2.2 LMCD1 depletion induces lipid accumulation and senescence in GC co‐incubated SCs

**FIGURE 2:**
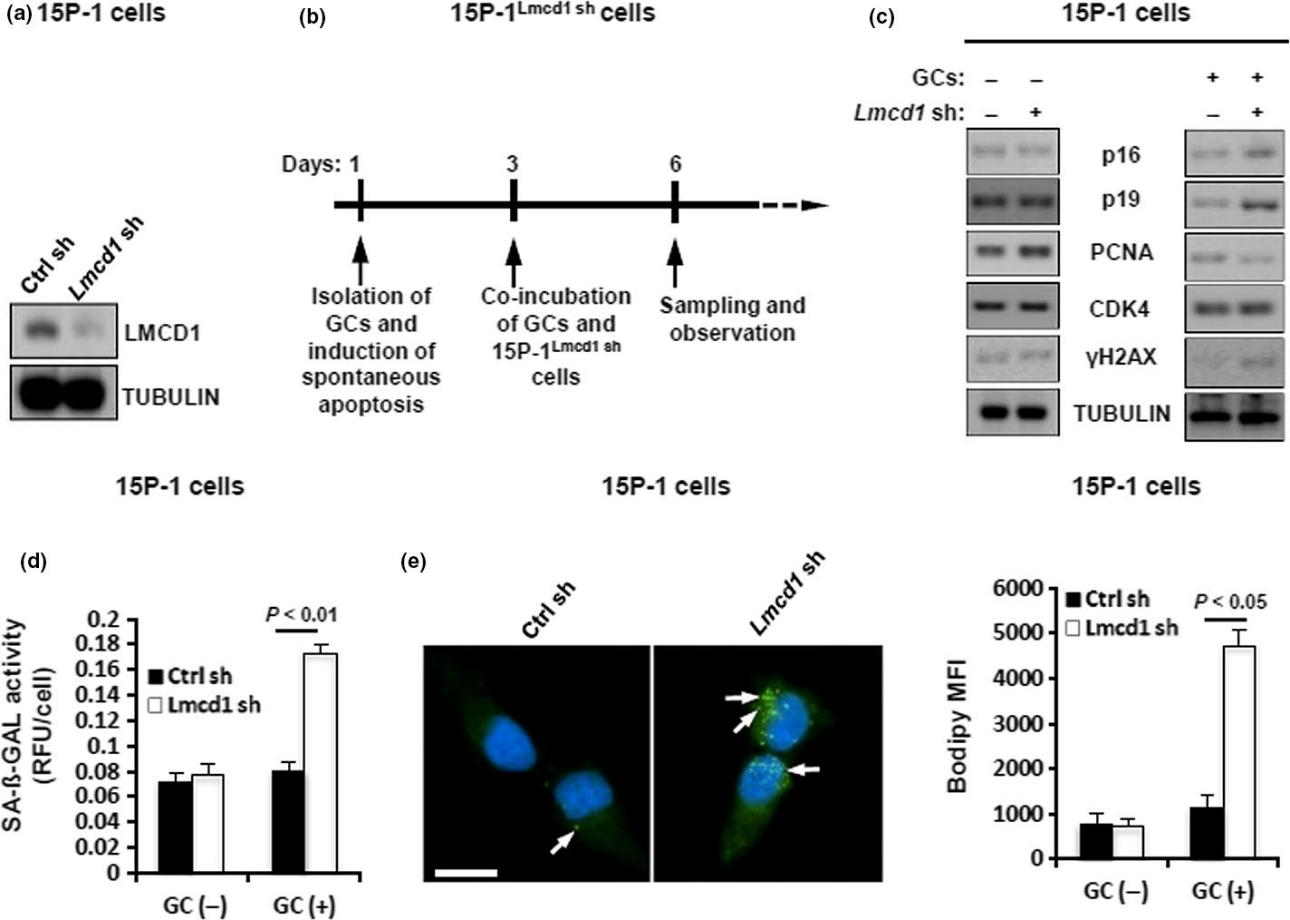
LMCD1 knockdown promotes lipid accumulation and senescence in GC co‐incubated SCs. (a) Generation of the 15P‐1 cells stably deprived of endogenous *Lmcd1* was verified using immunoblotting. (b) Schematic experimental design for senescence analysis after co‐incubation of GCs and 15P‐1^Lmcd1−/−^ cells. (c) Immunoblotting analysis of different senescence and proliferation markers in GC co‐incubated 15P‐1^Lmcd1−/−^ cells. (d) SA‐β‐gal activity analysis in GC co‐incubated 15P‐1^Lmcd1−/−^ cells. (e) Staining of GC co‐incubated 15P‐1 cells with BODIPY 493/503. Right panel, geometric mean fluorescence intensity (MFI) of BODIPY 493/503 in 15P‐1 cells was measured (*n* = 5)

The above data point to an association between LMCD1 deregulation and testicular aging. To address this, we generated the 15P‐1 cells that were stably deprived of endogenous *Lmcd1* expression (Figure 2a) and then subjected these cells to co‐incubation with apoptotic GCs. LMCD1 depletion caused upregulation of the cyclin‐dependent kinase inhibitors p16, p19, and damage‐related γH2AX, while proliferating cell nuclear antigen (PCNA) was reduced in GC co‐incubated 15P‐1^Lmcd1sh^ cells. These phenotypes were undetectable when 15P‐1^Lmcd1sh^ cells were cultured alone (Figure 2b,c). Consistent with these descriptive data, the GC co‐incubated 15P‐1^Lmcd1sh^ cells showed significantly increased SA‐ß‐GAL activity, suggesting stimulated senescence (Figure 2d). Because apoptotic GCs are phagocytosed and degraded by SCs to serve as an important energy sources for SCs (Xiong et al., 2009), and because lipid metabolism is tightly linked to cell senescence (Flor, Wolfgeher, Wu, & Kron, 2017), we sought to determine the evaluation of the amount of lipids in GC co‐incubated 15P‐1^Lmcd1sh^ cells using lipophilic fluorescent dye BODIPY 493/503. The GC co‐incubated 15P‐1^Lmcd1sh^ cells exhibited more fluorescence than their culture‐alone counterparts (Figure 2e).

### 2.3 Oxidative stress directly targets the *Lmcd1* transcription

**FIGURE 3:**
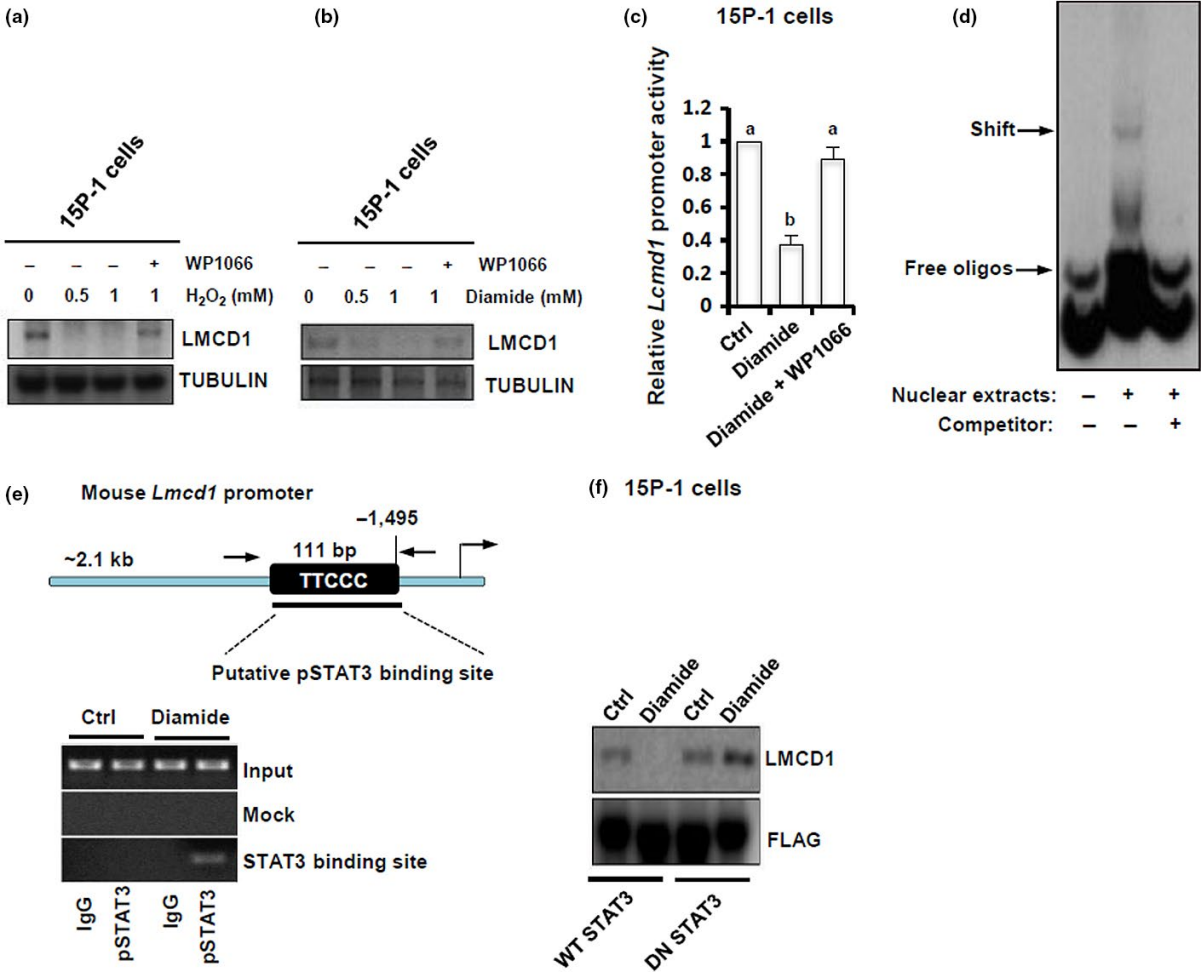
Oxidative stress directly regulates the Lmcd1 transcription. (a‐b) 15P‐1 cells were treated with different doses of H_2_O_2_ or diamide, in the presence or absence of co‐treatment with WP1066 (5 μM), for 6 h, followed by immunoblotting analysis. (c) 15P‐1 cells were transiently transfected with pGL4‐Lmcd1‐Luc. 48 h later, cells were treated with diamide (1 mM) or diamide plus WP1066 (5 μM) for 6 h, followed by the measurement of relative luciferase activity. Different superscript letters denote groups that are statistically different (*p* < 0.05). (d) 15P‐1 cells were transfected with Stat3 Flag pRc/CMV, which renders the STAT3 molecule constitutively active. 48 h later, 15P‐1 cells were treated with 1 mM of diamide for another 6 h, followed by nonradioactive electrophoretic mobility shift assay (EMSA). (e) ChIP analysis of pSTAT3 after diamide stimulation (1 mM) of the *Lmcd1* promoter. (f) 15P‐1 cells were transfected with WT STAT3 (Stat3 Flag pRc/CMV) or DN STAT3 (Stat3 Y705F Flag pRc/CMV) using Lipofectamine™ 3000. 48 h after transfection, cells were starved for 10 h and were then treated for 6 h with 1 mM of diamide, followed by immunoblotting analysis

Figure 1e also suggests that testicular oxidative stress may be involved in age‐related deregulation of LMCD1 expression. To directly address this, 15P‐1 cells were treated *in vitro* with 0.5 or 1 mM of H_2_O_2_ or oxidative stress inducer diamide for 6 h, and LMCD1 protein expression was determined. H_2_O_2_/diamide treatment inhibited LMCD1 expression in a dose‐dependent manner, whereas co‐treatment with WP1066 (an inhibitor of STAT3 activation) completely neutralized the inhibitory effects of H_2_O_2_/diamide on LMCD1 expression (Figure 3a,b). To ask whether the inhibition of LMCD1 expression by diamide was due to a direct transcriptional effect, 15P‐1 cells were transiently transfected with pGL4‐Lmcd1‐Luc and treated with diamide or diamide plus WP1066. While diamide caused a ~62.6% reduction in luciferase activity relative to control, co‐treatment with WP1066 completely restored the luciferase activity in diamide‐challenged cells (Figure 3c). Oxidative stress is known to trigger STAT3 tyrosine phosphorylation (Carballo et al., 1999). Our bioinformatic analysis revealed a putative STAT3‐binding site in the *Lmcd1* promoter. We next used EMSA to further examine direct binding of STAT3 onto the *Lmcd1* chromatin. STAT3 in the nuclear extracts from GC‐stimulated 15P‐1/STAT3 cells clearly formed a shifted DIG‐labeled band, which was totally abrogated by competition with a 50‐fold excess of cold probe (Figure 3d). To determine at the *in vivo* level whether STAT3 phosphorylation involved in oxidative stress regulation of *Lmcd1* transcription, we performed ChIP assay using anti‐pSTAT3. Treatment with diamide for 6 h in 15P‐1 cells stimulated a binding of pSTAT3 onto the *Lmcd1* promoter, whereas untreated cells exhibited no interaction (Figure 3e), indicating that diamide might repress *Lmcd1* transcription via STAT3 activation. Consistently, challenge with diamide for 6 h significantly impaired LMCD1 expression in WT STAT3‐transfected 15P‐1 cells but failed to affect LMCD1 expression in 15P‐1 cells transfected with dominant‐negative STAT3 (DN STAT3) (Figure 3f). Thus, functional STAT3 signaling is indispensable in oxidative stress's action to inhibit LMCD1 expression.

### 2.4 Requirement of endogenous Lmcd1 for murine spermatogenesis

**FIGURE 4:**
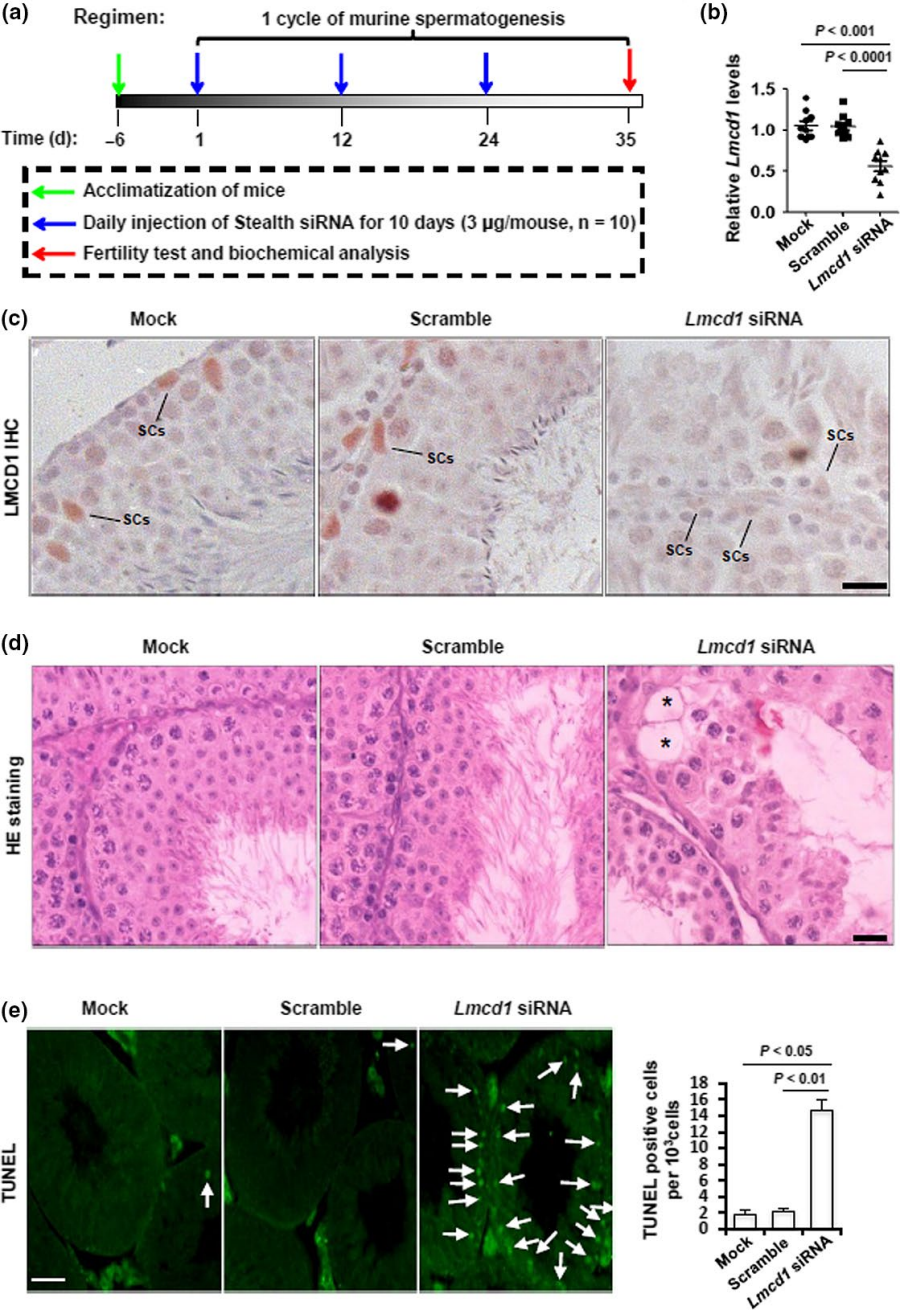
Effects of ablation of *Lmcd1* expression *in vivo* on testicular morphology and GC apoptosis. (a) Schematic representation of the experimental procedures used in the *in vivo* siRNA treatment. (b) Effects of endogenous *Lmcd1* knockdown in testis by *in vivo* siRNA treatment were confirmed by RT‐qPCR at transcriptional level. (c) Effects of endogenous *Lmcd1* knockdown in testis by *in vivo* siRNA treatment were confirmed by immunohistochemistry at translational level. Scale bar, 25 μm. (d) Effect of *in vivo* siRNA treatment on mouse testicular morphology was evaluated in H&E‐stained transverse testis sections. Scale bar, 25 μm. Asterisks denote vacuoles. (e) Effect of *in vivo* siRNA treatment on GC apoptosis was assessed using TUNEL staining, followed by the quantification of TUNEL‐positive cells (right panel)

To understand the biological effects of *Lmcd1*, we knocked down its expression *in vivo* using a previously validated protocol (Dong et al., 2016; Gonzalez‐Herrera et al., 2006). Treatment with *Lmcd1* Stealth siRNA as indicated (Figure 4a) caused a ~43.2% reduction in *Lmcd1* mRNA expression in testis compared with that in testis from Ctrl siRNA‐treated mice (Figure 4b). The effectiveness of siRNA treatment was further confirmed by immunostaining (Figure 4c). LMCD1 ablation resulted in more frequent and notable vacuoles and lack of mature sperms in the lumen of seminiferous tubules (Figure 4d). Moreover, the testes treated with *Lmcd1* siRNA exhibited a significantly higher level of apoptosis *in situ* (Figure 4e). In accordance with these morphological changes, *Lmcd1* knockdown significantly impaired fertility potential by inducing oligozoospermia and asthenospermia (Table S1). The integral *Lmcd1* expression is thus necessary for normal spermatogenesis.

### 2.5 Regulation of SC phagocytosis by TNF‐α/Lmcd1 signaling pathway

**FIGURE 5:**
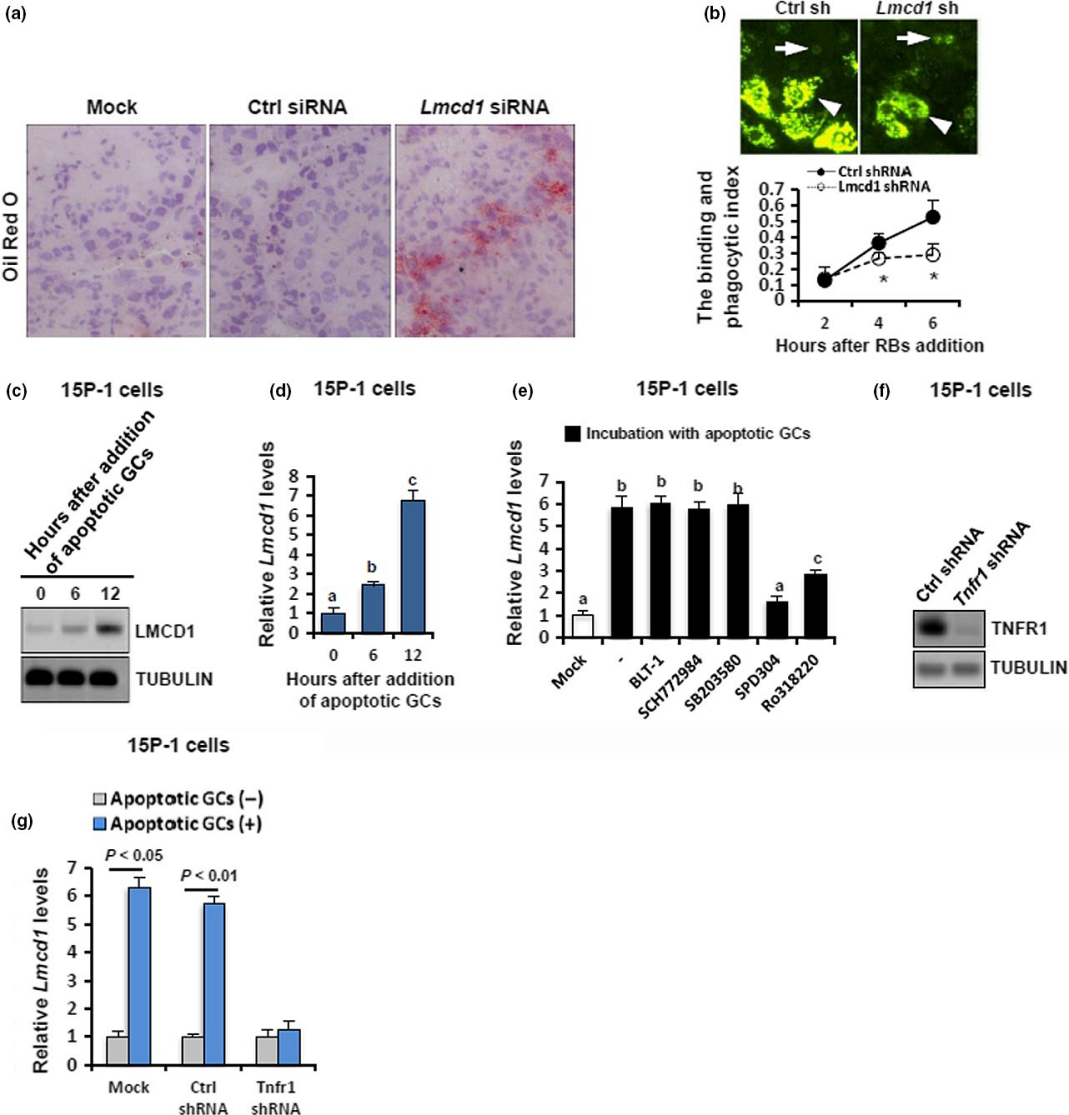
Functional relevance and paracrine control of LMCD1 expression during testicular phagocytosis. (a) Effect of siRNA treatment on testicular phagocytosis *in vivo* was evaluated using Oil Red O staining. (b) Upper panel: Representative pictures of residual body (RB) binding and phagocytized by Ctrl siRNA or *Lmcd1* siRNA‐treated 15P‐1 cells 6 h after addition of RBs. Arrowheads denote the ingested and plasma membrane‐bound RBs, whereas arrows denote the cells with no phagocytized RBs. Lower panel: Kinetics of RB binding and phagocytosis by SCs (the binding and phagocytosis index) were calculated as the ratio of positive green signals/nuclei number in each well. Quantitative values were presented as mean ± *SD* of three independent experiments (*p < 0.05). (c) Wild‐type 15P‐1 cells were incubated with apoptotic GCs for different durations as indicated, followed by immunoblotting analysis. (d) Wild‐type 15P‐1 cells were incubated with apoptotic GCs for different durations as indicated, followed by RT‐qPCR analysis. Different superscript letters denote groups that are statistically different (*p* < 0.05). (e) RT‐qPCR showing *Lmcd1* expression in 15P‐1 cells induced with apoptotic GCs in the presence or absence of different pathway inhibitors. Different superscript letters denote groups that are statistically different (*p* < 0.05). (f) Generation of the 15P‐1 cells stably deprived of endogenous *Tnfr1* was verified using immunoblotting. (g) The 15P‐1 cells with different transfections were treated with apoptotic GCs for 6 h, followed by RT‐qPCR analysis

Because efficient phagocytic clearance of apoptotic GCs by SCs is critical for homeostasis inside the seminiferous tubules (Dong et al., 2016), we were curious whether the abnormal accumulation of testicular apoptosis is due to a potential impairment in phagocytosis. Deregulated metabolism of the lipid droplets is a hallmark of disrupted phagocytic function in SCs (Dong et al., 2016). As shown by Oil Red O staining, lipid droplet accumulation was dramatically increased in *Lmcd1* siRNA‐treated testis compared with a negligible staining in mock and Ctrl siRNA‐treated testes (Figure 5a), indicative of a phagocytic disturbance upon LMCD1 deficiency. A 6‐h incubation with biotin‐labeled residual bodies (RBs) stimulated a steady increase in the binding and phagocytic activities in Ctrl shRNA‐treated 15P‐1 cells, but not in 15P‐1^Lmcd1−/−^ cells (Figure 5b). Consistently, incubation of 15P‐1 cells with apoptotic GCs elicited a gradual increase in LMCD1 expression in a time‐dependent manner, at both protein (Figure 5c) and mRNA (Figure 5d) levels. To ask whether other pathways integrate the GC‐LMCD1 interaction, we stimulated 15P‐1 cells with apoptotic GCs in the presence of different pathway inhibitors. Concomitant incubation with SPD304 and Ro318220 substantially reduced the *Lmcd1* induction by GCs (Figure 5e), suggesting that *Lmcd1* upregulation during phagocytosis may be dependent on TNF‐α/PKC activation. To further determine the requirement of TNF‐α signaling for GC‐induced *Lmcd1* expression, we stably knocked down the TNFR1 expression in 15P‐1 cells (Figure 5f). Consequently, the deprivation of TNFR1 successfully abolished apoptotic GC‐induced *Lmcd1* expression in 15P‐1 cells (Figure 5g). Collectively, TNF‐α signaling may regulate *Lmcd1* induction during phagocytosis.

### 2.6 Establishment of TXLNA as the main downstream effector of Lmcd1 signaling during phagocytosis

**FIGURE 6:**
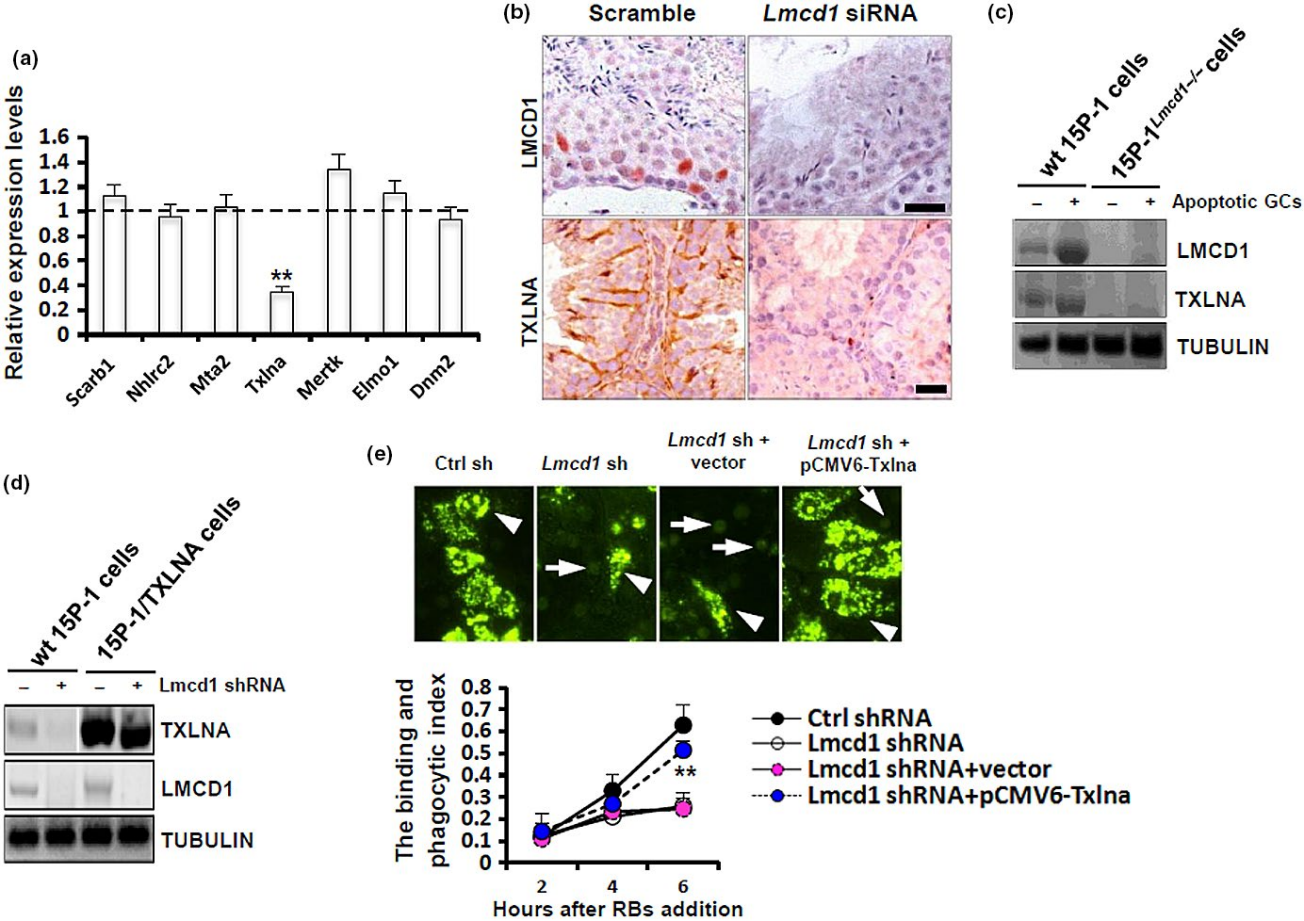
*Lmcd1* depletion compromises TXLNA‐mediated testicular phagocytosis. (a) Expression levels of different key factors essential for testicular phagocytosis in *Lmcd1* siRNA‐treated testis were determined using RT‐qPCR. **p < 0.01 compared with the values in Ctrl siRNA‐treated testis. (b) Expression levels of LMCD1 and TXLNA in *Lmcd1* siRNA‐treated testis were evaluated using immunohistochemistry at translational level. Scale bar, 20 μm. (c) Wild‐type 15P‐1 and 15P‐1^Lmcd1−/−^ cells were incubated with apoptotic GCs for 6 h, followed by immunoblotting analysis. (d) Wild‐type 15P‐1 and 15P‐1/TXLNA cells were transduced with *Lmcd1* shRNA for 48 h, followed by immunoblotting analysis. (e) Wild‐type 15P‐1 and 15P‐1^Lmcd1−/−^ cells were transiently transfected with pCMV6‐Txlna or empty vectors. 48 h later, cells were subjected to the RB binding and phagocytosis assay, as described above. **p < 0.01 comparing Lmcd1 shRNA+vector to Lmcd1 shRNA+pCMV6‐Txlna

As further exploration of the molecular basis underpinning *Lmcd1* action, we investigated the expression levels of several key factors known to be essential for SC phagocytosis, in Stealth siRNA‐treated testis using RT‐qPCR. Interestingly, *Lmcd1* inhibition was accompanied by decreased *Txlna* expression by ~62.4% in Stealth siRNA‐treated testis (Figure 6a). This impaired TXLNA expression was further confirmed at the protein level by immunostaining (Figure 6b). In keeping with this, co‐incubation with apoptotic GCs for 6 h failed to stimulate TXLNA expression in 15P‐1^Lmcd1−/−^ cells (Figure 6c). We then determined whether TXLNA alone was sufficient to explain *Txlna* deficiency‐caused phagocytic disturbance. As expected, transient transfection of 15P‐1/TXLNA cells with *Lmcd1* shRNA significantly reduced LMCD1 expression but had no effects on TXLNA levels (Figure 6d). More importantly, TXLNA overexpression successfully ameliorates the phagocytic activities by 15P‐1 cells in the presence of LMCD1 inhibition (Figure 6e). Thus, these findings confirm the relevance of the disruption of the LMCD1/TXLNA pathway in conferring phagocytic disturbance.

### 2.7 Requirement of dephosphorylation of NFAT1 by LMCD1 for TXLNA activation in SCs

**FIGURE 7:**
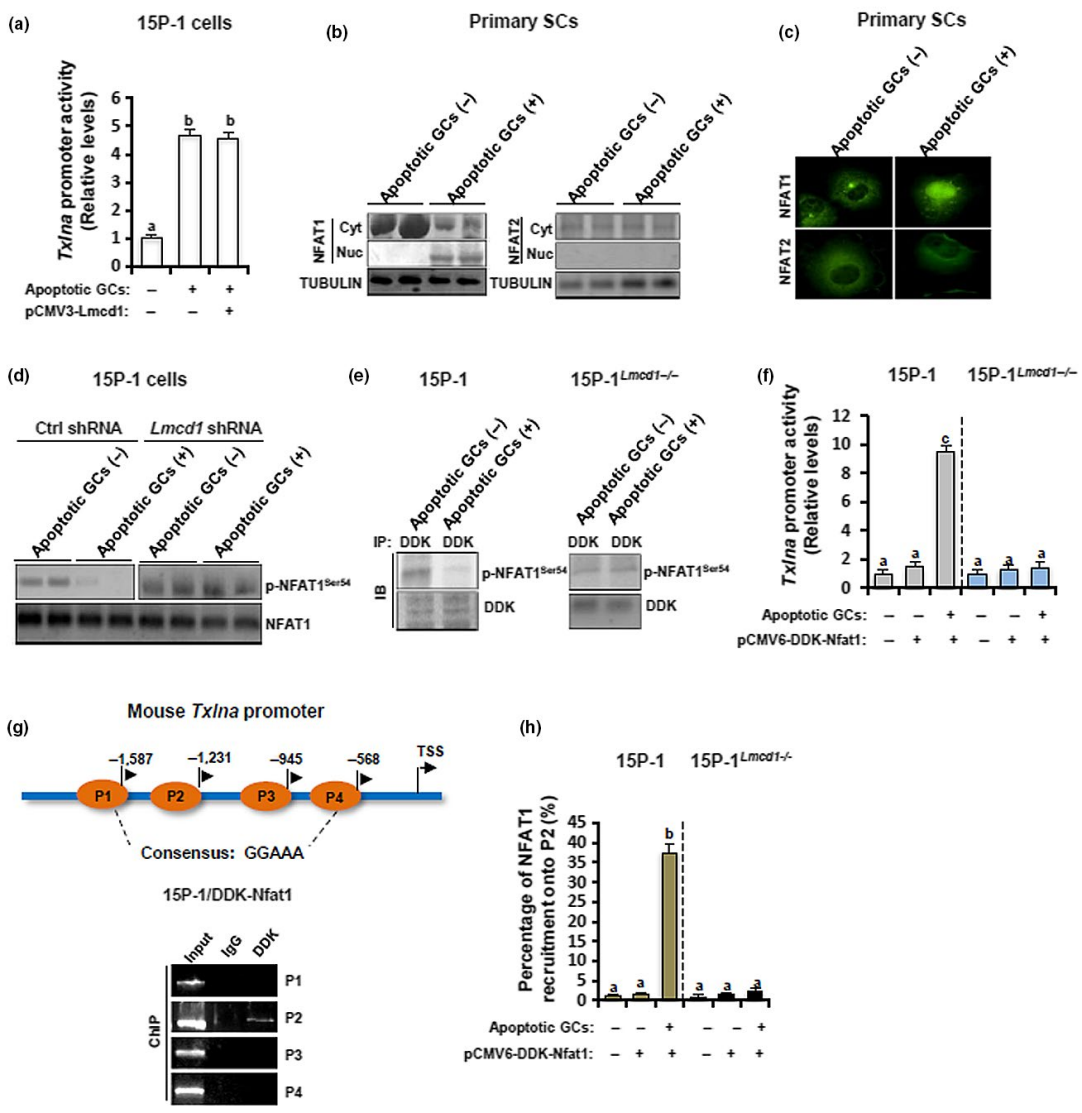
LMCD1 regulates the dephosphorylation and nuclear translocation of NFAT1, which consequently expedites the transactivation of *Txlna*, a binding partner of the syntaxin family that functions as a central regulator of testicular phagocytosis. (a) 0.5 μg reporter plasmid and pRL‐TK Renilla reporter plasmid were co‐transfected into 15P‐1 cells with different plasmids as indicated. 48 h later, cells were incubated with apoptotic GCs for 6 h, followed by the measurement of luciferase activities using a dual‐luciferase reporter system from Promega. Different superscript letters denote groups that are statistically different (*p* < 0.05). (b) Primary SCs were incubated with apoptotic GCs for 6 h. Cells were then subjected to nuclear and cytoplasmic fractionation and immunoblotting analysis. (c) Primary SCs were incubated with apoptotic GCs for 6 h, followed by immunofluorescence. (d) Wild‐type 15P‐1 and 15P‐1^Lmcd1−/−^ cells were incubated with apoptotic GCs for 6 h, followed by immunoblotting analysis. (e) Wild‐type 15P‐1 and 15P‐1^Lmcd1−/−^ cells were transiently transfected with pCMV6‐DDK‐Nfat1. 48 h later, cells were incubated with apoptotic GCs for 6 h, followed by Co‐IP analysis. (f) 0.5 μg reporter plasmid and pRL‐TK Renilla reporter plasmid were co‐transfected into wild‐type 15P‐1 and 15P‐1^Lmcd1−/−^ cells with different plasmids as indicated. 48 h later, cells were incubated with apoptotic GCs for 6 h, followed by the measurement of luciferase activities using a dual‐luciferase reporter system from Promega. Different superscript letters denote groups that are statistically different (*p* < 0.05). (g) The 15P‐1/DDK‐Nfat1 cells were incubated with apoptotic GCs for 6 h, followed by ChIP assay using anti‐DDK antibody. (h) Wild‐type 15P‐1 and 15P‐1^Lmcd1−/−^ cells were transfected with pCMV6‐DDK‐Nfat1. 48 h later, cells were incubated with apoptotic GCs for 6 h, followed by ChIP‐qPCR analysis using anti‐DDK antibody. Different superscript letters denote groups that are statistically different (*p* < 0.05)

Because LMCD1 functions at the transcriptional level (Du et al., 2016), we initially hypothesized that LMCD1 may directly regulate the *Txlna* transcription, but apparently this was not the case since transfection with pCMV3‐Lmcd1 failed to potentiate the apoptotic GC‐induced transactivation of *Txlna* in 15P‐1 cells (Figure 7a). The calcium‐dependent pathway plays an essential regulatory role along germ cell development (Yuan et al., 2006). A recent study has shown that LMCD1 regulates cardiac hypertrophy by targeting calcium‐related NFAT signaling (Bian et al., 2010). These findings made us curious about a potential involvement of NFATs in SC function. In resting cells, NFATs are normally localized in the cytoplasm due to hyperphosphorylation. Instead, upon different stimulations, NFAT proteins are dephosphorylated, thus translocating to the nucleus to activate target gene expression (Sharma et al., 2011). Interestingly, co‐incubation of primary SCs with apoptotic GCs for 6 h only promoted the translocation of NFAT1 to the nuclei but had no effects on the cytoplasmic localization of NFAT2 (Figure 7a,b). Consistently, expression levels of p‐NFAT1^Ser54^ were significantly attenuated in the 15P‐1 cells treated with apoptotic GCs, whereas this dephosphorylation of NFAT1 was completely abrogated in 15P‐1^Lmcd1−/−^ cells treated with apoptotic GCs (Figure 7c). To unambiguously demonstrate the requirement of LMCD1 in the dephosphorylation of NFAT1, we transfected 15P‐1 and 15P‐1^Lmcd1−/−^ cells with pCMV6‐DDK‐Nfat1 and then stimulated these cells with apoptotic GCs for 6 h. Subsequent Co‐IP assay using anti‐DDK antibody revealed that the exogenous NFAT1 was hypophosphorylated in wild‐type 15P‐1 cells but was hyperphosphorylated in 15P‐1^Lmcd1−/−^ cells, upon GC stimulation (Figure 7d). To ask whether NFAT1 directly targeted the *Txlna* chromatin, we performed luciferase reporter assay. In wild‐type 15P‐1 cells treated with apoptotic GCs, NFAT1 directly bound to the promoter of *Txlna*, and by which it remarkably increased luciferase activities. By contrast, 15P‐1^Lmcd1−/−^ cells co‐transfected with pGL4‐Luc‐*Txlna* and pCMV6‐DDK‐Nfat1 demonstrated unaffected luciferase activities (Figure 7e). To demonstrate the occupancy of NFAT1 onto the *Txlna* chromatin *in vivo*, we carried out ChIP assay in 15P‐1/DDK‐Nfat1 cells. NFAT1 could directly bind the promoter region (P2) that harbored the consensus binding sequence "GGAAA" in the presence of GC stimulation (Figure 7f). Subsequent ChIP‐qPCR analysis showed that the recruitment of NFAT1 onto *Txlna* promoter only occurred in GC‐treated wild‐type 15P‐1 cells, but was undetectable in resting 15P‐1 cells or in 15P‐1^Lmcd1−/−^ cells treated with apoptotic GCs (Figure 7g,h). Taken together, these results suggest that the potentiation of *Txlna* transactivation by NFAT1 requires the integral LMCD1 expression in phagocytic SCs.

### 2.8 Rescuing effects of overexpression of a constitutively active NFAT1 on LMCD1 deficiency‐induced phagocytic disturbance

**FIGURE 8:**
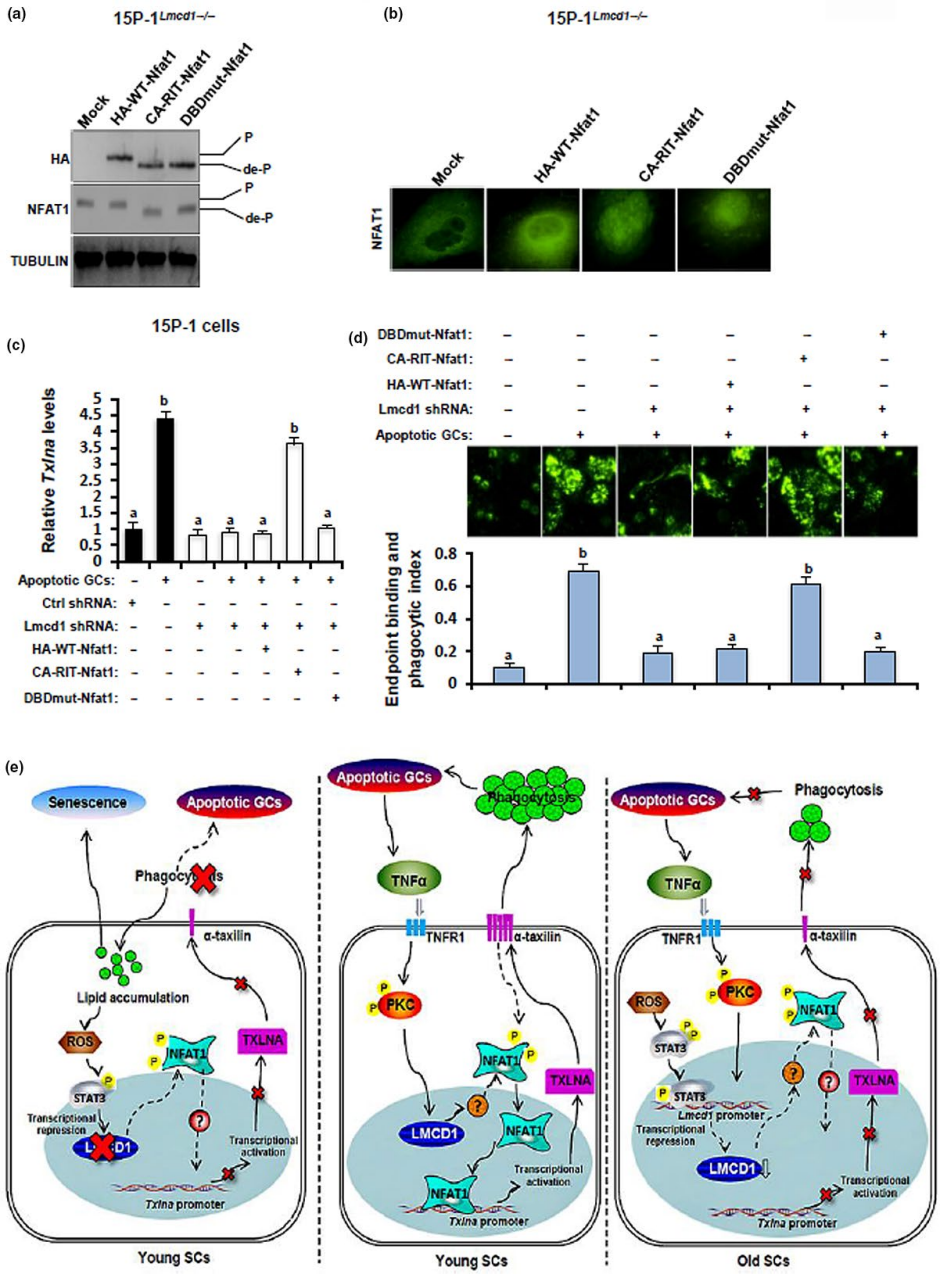
Overexpression of a constitutively active NFAT1 ameliorates the LMCD1 deficiency‐impaired *Txlna* expression and phagocytic activities in 15P‐1 cells. (a) 15P‐1^Lmcd1−/−^ cells were transfected with different mutant plasmids as described in the EXPERIMENTAL PROCEDURES section, followed by immunoblotting. (b) 15P‐1^Lmcd1−/−^ cells were transfected with different mutant plasmids as described in the EXPERIMENTAL PROCEDURES section, followed by immunofluorescence. (c) 15P‐1^Lmcd1−/−^ cells with different transfections were incubated with apoptotic GCs for 6 h, followed by the measurement of *Txlna* expression using RT‐qPCR. Different superscript letters denote groups that are statistically different (*p* < 0.05). (d) 15P‐1^Lmcd1−/−^ cells with different transfections were subjected to the RB binding and phagocytosis assay, as described above. Different superscript letters denote groups that are statistically different (*p* < 0.05). (e) Proposed working model by the current study: SC‐specific expression of LMCD1 is under the precise control of apoptotic GC‐produced TNF‐α signaling. LMCD1 facilitates the dephosphorylation and nuclear translocation of NFAT1, which consequently potentiates the *Txlna* transactivation and thus promotes the removal of apoptotic GCs by phagocytic functions of SCs. Disruption in phagocytosis by LMCD1 deficiency causes lipid accumulation, increase in oxidative stress, and cellular senescence in phagocytic SCs

The preceding data made us wonder whether disturbance in the dephosphorylation and nuclear translocation of NFAT1 alone could explain for the deleterious effects of Lmcd1 deficiency on the TXLNA‐mediated phagocytic activities. To address this, 15P‐1^Lmcd1−/−^ cells were transduced with bicistronic retrovirus (IRES‐GFP or IRES‐Thy1.1) retroviruses: empty (mock), HA‐tagged wild‐type Nfat1 (HA‐WT‐Nfat1), and HA‐tagged constitutively active Nfat1 with the RIT mutation that to interfere selectively with the NFAT:AP‐1 interaction (CA‐RIT‐Nfat1) and HA‐tagged constitutively active Nfat1 with RIT mutation as well as a mutation in the DNA‐binding domain that prevents NFAT1 DNA binding (DBDmut‐Nfat1). As shown by immunoblotting assay, HA‐tagged CA‐RIT‐Nfat1 and DBDmut‐Nfat1 migrate at a lower apparent molecular weight than HA‐tagged WT‐Nfat1, reflecting the fact that CA‐RIT‐Nfat1 and DBDmut‐Nfat1 mimic dephosphorylated NFAT1. In line with the previous report (Martinez et al., 2015), WT, CA‐RIT‐Nfat1, and DBDmut‐Nfat1 were not massively overexpressed relative to endogenous NFAT1 in 15P‐1^Lmcd1−/−^ cells (Figure 8a). Moreover, CA‐RIT‐Nfat1 and DBDmut‐Nfat1 were both observed to be localized in nuclei, and this nuclear localization was undetectable in mock or HA‐WT‐Nfat1‐transfected cells (Figure 8b). Importantly, compared to cells transduced with mock, HA‐WT‐Nfat1, or DBDmut‐Nfat1, cells expressing CA‐RIT‐Nfat1 successfully ameliorated LMCD1 deficiency‐impaired expression of *Txlna* mRNA in GC‐challenged 15P‐1^Lmcd1−/−^ cells (Figure 8c). Accordingly, ectopic CA‐RIT‐Nfat1 expression completely rescued LMCD1 deficiency‐attenuated phagocytic activities, whereas transduction with HA‐WT‐Nfat1 or DBDmut‐Nfat1 had no any effects on phagocytic activities, in GC‐challenged 15P‐1^Lmcd1−/−^ cells (Figure 8d). Thus, the disturbance in the dephosphorylation and nuclear translocation of NFAT1 mediates, at least in part, LMCD1 deficiency‐impaired phagocytosis in SCs.

## 3 DISCUSSION

By using old testis and 15P‐1^Lmcd1−/−^ cells, we firstly found that LMCD1 deficiency promoted cellular senescence in SCs, likely as a result of lipid accumulation. In support of our hypothesis, deregulation of lipid metabolism has been shown to potentiate senescence in many cell lines including retinal epithelial cells (Chen et al., 2019), endothelial cells (Nannelli et al., 2018), and cancer cells (Flor et al., 2017). Of note, the promotion of cellular senescence was only detectable in the GC‐incubated 15P‐1^Lmcd1−/−^ cells but not in 15P‐1^Lmcd1−/−^ cells alone, suggesting that the LMCD1 action in SCs may require interaction with adjacent GCs. In this regard, the promotion effect of LMCD1 deficiency on SC senescence may be attributed to the disruption in phagocytic function of SCs, but unlikely due to metabolic disturbance in SCs themselves. In favor of our ratiocination, previous studies using neutrophils and macrophages have shown that a failure to mount an efficient autophagy, a similar phagocytic process inside cells, is a deviation on the cell's part from normal cellular function into cell senescence and cessation of the cell cycle (Gomez‐Cambronero & Kantonen, 2014). Therefore, such a LMCD1 deficiency‐based cellular senescence mechanism may possibly involve two sequential inputs: impaired phagocytosis and ineffectiveness in clearing away decomposed GC fragments that ultimately lead to lipid accumulation (Figure 8e).

Testicular dysfunction caused by increased oxidative stress is a hallmark of aging males (Huang et al., 2018). Nevertheless, whether oxidative stress damages male fertility through affecting testicular phagocytosis remains unknown. We have shown that oxidative stress negatively regulated *Lmcd1* transcription through a STAT3‐dependent pathway in SCs. Because ROS‐mediated activation of STAT3 signaling is involved in stress‐induced cellular senescence (Liu et al., 2017), and because ROS/STAT3 signaling pathway also regulates autophagy of cancer cells (Yoon et al., 2010), we reason that LMCD1 may function as an important converging point linking oxidative stress, testicular phagocytosis, and SC senescence. Moreover, since disruption of lipid homeostasis is well known for induction of the increased oxidant species in senescent cells (Chen et al., 2019) and apoptotic GCs are phagocytosed and degraded into lipids by SCs to fulfill energy requirement (Xiong et al., 2009), a reciprocal feedback loop is very likely to exist between ROS/LMCD1 signaling and lipid‐elicited oxidative stress in phagocytic SCs.

The regulation of testicular LMCD1 expression by SR‐BI and TNF‐α was assessed, given their major roles in the control of testicular phagocytosis (Shiratsuchi, Kawasaki, Ikemoto, Arai, & Nakanishi, 1999) and SC function (Pentikainen et al., 2001), respectively. Activation of p38 and ERK1/2 by SR‐BI is necessary for a maximal level of testicular phagocytosis (Osada, Sunatani, Kim, Nakanishi, & Shiratsuchi, 2009). TNF‐α, a pleiotropic cytokine produced by GCs, binds to TNFR1 and mediates multiple biologic responses in SCs including androgen receptor expression (Delfino, Boustead, Fix, & Walker, 2003), production of lactate (Riera et al., 2001), immune homeostasis (Wu et al., 2016), and SC apoptosis (Wang & Su, 2018). Accumulating data evidence that protein kinase C (PKC) is one of the core intracellular mediators of TNF‐α actions (Chang & Tepperman, 2001). Intriguingly, our results showed that co‐incubation with either TNF‐α inhibitor SPD304 or PKC inhibitor Ro318220 could both effectively abolish the GC‐elicited upregulation of *Lmcd1* transcription. The ability of TNF‐α to acutely stimulate *Lmcd1* mRNA was further confirmed using the 15P‐1^Tnfr1−/−^ cells, which demonstrated also that TNFR1 depletion totally abrogated dead GC‐induced *Lmcd1* mRNA expression. Overall, our current results strongly suggest that SC‐specific expression of LMCD1 is subjected to precise regulation, involving a paracrine control by GC‐released TNF‐α.

Further evidence for an essential regulation of male fertility by LMCD1 through control of SC phagocytosis is provided by the promoting effects of LMCD1 depletion on the accumulation of lipid droplets in SCs and by the deleterious effects of LMCD1 depletion on phagocytic activities *in vitro*. To note, unlike other key factors regulating phagocytosis in SCs (Elliott et al., 2010), mice treated with *Lmcd1* Stealth siRNAs *in vivo* only displayed impaired male infertility but not absolute sterility. One tempting possibility is that our *in vivo* siRNA approach only partially inhibited but not totally depleted the LMCD1 expression in SCs. Further experimental work, including employment of a Sertoli‐specific Lmcd1 KO mouse, is needed to fully elucidate the functions of LMCD1 in SCs.

Ca^2+^/calmodulin‐dependent pathways are essentially involved in the regulation of testicular functions under both physiological and pathological conditions. The nuclear factor of activated T cells (NFAT) family is such a striking example. NFAT2 is implicated in corticosterone‐induced rat Leydig cell apoptosis (Chai, Wang, & Gao, 2007). NFAT1‐regulated anti‐inflammatory response exerts a potent protective effect on testicular macrophages upon infection with uropathogenic E. coli (Bhushan et al., 2011). Nevertheless, the functional details of distinct NFAT members in different testicular cell types and corresponding molecular basis remain poorly understood. Our findings expand this knowledge by identifying NFAT1 as a downstream effector of LMCD1 pathway in SCs. Facilitation of the nuclear translocation of NFAT1 via potentiating its dephosphorylation, thereafter promoting the NFAT1‐mediated transactivation of *Txlna*, may represent a novel mechanism contributing to the maintenance of SC phagocytosis. Multiple lines of observations may substantiate this hypothesis: (a) Using Western blotting and immunofluorescence, we have shown that NFAT1 was the major NFAT isoform translocating into nuclei during phagocytosis; (b) both endogenous and exogenous NFAT1 proteins in SCs were dephosphorylated upon treatment with apoptotic GCs; (c) by using ChIP and luciferase reporter assays, we demonstrated that in the presence of LMCD1 expression, NFAT1 could directly bind the promoter region of *Txlna*, thus potentiating its transactivation in phagocytic SCs; and (d) most importantly, LMCD1 deficiency‐attenuated *Txlna* expression and phagocytic activities could be successfully ameliorated by ectopic expression of a constitutively active NFAT1 mutant (CA‐RIT‐Nfat1) in SCs. Given that LIM domain proteins regulate diverse biological processes mainly through interacting or binding of target proteins (Bian et al., 2010), the available data thus strongly suggest that LMCD1 functions as an essential transcriptional cofactor that helps maintain the function of NFAT1/*Txlna* cascade during phagocytosis. In favor of this, it has been shown that LMCD1 inhibits DNA binding of transcriptional factor GATA6 and thus restricts its function during lung and heart development (Rath, Wang, Lu, & Morrisey, 2005). Likewise, by acting as a co‐activator with E2F transcription factor 1, LMCD1 stimulates human aortic smooth muscle cell (HASMC) proliferation and thereby promotes human atherogenesis (Janjanam et al., 2018). In this context, a precise balance between interactions with LMCD1 and NFAT1 is indispensable for the regulation of spatial and temporal activity of NFAT1 as well as other key transcriptional factors in phagocytic SCs.

In summary, our current results unambiguously demonstrate that SC‐specific expression of LMCD1 is essential for phagocytic activity and this expression is under the precise control of both apoptotic GC‐produced TNF‐α and lipid‐elicited ROS signaling pathways. One major mechanism underlying the LMCD1 action originates from its ability to facilitate the dephosphorylation and nuclear translocation of NFAT1, which consequently expedites the *Txlna* transactivation and thus promotes the removal of apoptotic GCs by phagocytic function of SCs (Figure 8e). Overall, LMCD1 may operate as a novel pretranscriptional integrator linking SC phagocytosis, lipid homeostasis, and cell senescence.

## 4 EXPERIMENTAL PROCEDURES

### 4.1 Animal

Male C57BL/6J mice were obtained from the Animal Research Center of our university. Mice were *fed ad libitum* and were allowed to acclimatize for at least 1 week before the experiment. Mice were finally euthanized by CO_2_ inhalation followed by cervical dislocation. The procedures involved in the animal work, strictly conformed to the *Guide for the Care and Use of Laboratory Animals published by the National Institutes of Health*, were approved by the Animal Care and Use Committee of Fourth Military Medical University (KY20183306‐1).

To study oxidative stress regulation of LMCD1 expression, 540‐day‐old mice received i.p. injections with EGCG (50 mg/kg, MedChemExpress, Shanghai, China) for consecutive 3 days, followed by a 4‐day break. Mice received a total of 4 cycles of EGCG treatment.

*In vivo* ablation of endogenous LMCD1 expression in testis was achieved using a previously established protocol (Dong et al., 2016; Gonzalez‐Herrera et al., 2006). Briefly, mice were injected intraperitoneally (i.p.) with 100 μl solution of Lmcd1 Stealth siRNAs or scramble Ctrl siRNAs daily for 10 days in isotonic saline solution (3 μg/mouse) followed by 2 days of break. Mice received a total of three cycles of siRNA injections (~35 days), and this duration was selected because one cycle of murine spermatogenesis consists of 35 days (Timmons, Rigby, & Poirier, 2002). At the end of 35 days after the first siRNA injection, mice were subjected to the evaluation of reproductive capacity and characterization of epididymal sperms status, as described in detail in our previous work (Dong et al., 2016).

### 4.2 Cell treatment

15P‐1 cells were obtained from the American Type Culture Collection (Manassas, VA, USA) and maintained in DMEM/F12 containing 10% fetal calf serum (Thermo Fisher Scientific, Shanghai, China) in a humidified atmosphere of 5% CO_2_ at 37°C. Primary germ cells (GCs), SCs, and caudal sperms were isolated and purified from 8‐week‐old male C57BL/6J mice, as described by our previous work (Li et al., 2011; Ma et al., 2010; Zhang et al., 2013). GCs were cultured alone for 2 days to induce spontaneous apoptosis (Wang et al., 2006). To answer whether moderate oxidative stress regulated LMCD1 expression, 15P‐1 cells were treated with different doses of H_2_O_2_ or diamide (Sigma‐Aldrich, Shanghai, China), in the presence or absence of co‐treatment with WP1066 (5 μM; Selleck, Shanghai, China), for 6 h. 15P‐1 cells were transfected with WT STAT3 (Stat3 Flag pRc/CMV) or DN STAT3 (Stat3 Y705F Flag pRc/CMV) (Addgene, Watertown, MA, USA) using Lipofectamine™ 3000 (Thermo Fisher Scientific). 48 h after transfection, cells were starved for 10 h and were then treated for 6 h with 1 mM of diamide, followed by immunoblotting analysis. The 15P‐1 cells stably deprived of endogenous *Lmcd1* or *Tnfr1* were established by transfection using Lipofectamine® 3000 (Thermo Fisher Scientific) with *Lmcd1* shRNA or *Tnfr1* shRNA, along with the corresponding Ctrl shRNA, followed by selection with 0.5 μg/ml of G418 (Sigma‐Aldrich). 15P‐1/TXLNA cells that stably expressed exogenous mouse Txlna were generated by transfection with pCMV6‐Txlna or empty vector (OriGene, Beijing, China) followed by selection with 0.5 μg/ml of G418. To transiently express the exogenous mouse Nfat1, cells were transfected with pCMV6‐DDK‐Nfat1 or empty vector (Sino Biological, Beijing, China) using Lipofectamine® 3000 for 48 h, prior to other assays. Retroviral plasmids that expressed the wild‐type mouse Nfat1 (WT‐Nfat1), a constitutively active mouse Nfat1, mutated to interfere selectively with the NFAT:AP‐1 interaction (CA‐RIT‐Nfat1), and a constitutively active mouse Nfat1 that is unable to interact with AP‐1 with four‐point mutations in the DNA‐binding loop that abolish DNA binding (DBDmut‐Nfat1) were all gifts from Anjana Rao (Addgene plasmid #11100, Addgene plasmid #63214, and Addgene plasmid #63669; Addgene, Watertown, MA, USA). The 15P‐1 cells that stably expressed different exogenous mouse Nfat1 were generated by transfected with the aforementioned plasmids followed by selection with 50 ng/ml of puromycin (Sigma‐Aldrich) or by fluorescence‐activated cell sorting, respectively. To study the potential involvement of different pathways in apoptotic GC‐induced Lmcd1 expression, 15P‐1 cells were incubated with apoptotic GCs, in the presence of different pathway inhibitors including 5 μM of BLT‐1 (SR‐BI), 0.5 μM of SCH772984 (ERK1/2), 5 μM of SB203580 (p38 MAPK), 1 μM of SPD304 (TNF‐α), and 5 μM of Ro318220 (PKC), for 6 h, followed by RT‐qPCR analysis. BLT‐1 was from Merck (Shanghai, China), and the other inhibitors were from MedChemExpress (Shanghai, China).

### 4.3 In vitro binding and phagocytosis assay

RBs were isolated and purified from testes of 8‐week‐old mice, as described in previous publications (Dong et al., 2016; Yefimova et al., 2008). In brief, seminiferous tubules were digested by in 25 ml of 0.01 M PBS (pH 7.2) containing 0.1% glucose, 3 mM lactate, and 0.25 mg/ml trypsin for 20 min at 33°C. The resultant cell suspension was filtered through sterile surgical gauze, followed by three consecutive centrifugations at 800 *g* for 10 min. The resultant supernatants that contained RBs were stored at 4°C and were then subjected to biotin labeling using an EZ‐Link NHS‐PEG4 Biotinylation Kit (Thermo Fisher Scientific). To measure the phagocytotic activity *in vitro*, 15P‐1 cells were seeded in eight‐well Labtek chambers and were then incubated with 1.2 × 10^7^ biotinylated RBs at 33°C in a humidified atmosphere of 5% CO2 for different durations as indicated. Upon completion of incubation, unbound RBs were washed away with culture medium and fixed for 5 min with 4% paraformaldehyde/PBS (pH 7.4), followed by labeling with avidin‐FITC at 25°C for another 1 h. The ingested and membrane‐bound RBs were observed using an inverted microscope (Axio Imager M1 Microscope, Zeiss), and the binding and phagocytic index was calculated as the ratio of positive green signals/cell number in each well.

Detailed information on histological examination, measurement of testicular oxidative stress, lipid accumulation and cellular senescence determination, RT‐qPCR, immunoblotting, luciferase reporter assay, co‐immunoprecipitation (Co‐IP) and chromatin immunoprecipitation (ChIP), nonradioactive electrophoretic mobility shift assay (EMSA), and statistical analysis pertinent to this paper can be found in Supporting Information.

## CONFLICT OF INTEREST

The authors declare no conflict of interest.

## AUTHOR CONTRIBUTIONS

WL and YZ designed and supervised the experiments, contributed reagents, and analyzed the data. WL wrote and revised the paper and supervised the submission. XJ and SZ completed the majority of experiments and analyzed the data, and XJ wrote the manuscript. TD, PZ, and CZ performed the experiments and helped to analyze the data.

## Supporting information

Fig S1

Fig S2

Table S1

Table S2

Table S3

Supplementary Material

Fig S1‐S2‐Cap
