# Supplementary figures and images for: Testicular Lmcd1 regulates phagocytosis by Sertoli cells through modulation of NFAT1/Txlna signaling pathway
Source: Aging Cell. 2020 Aug 9;19(10):e13217. doi: 10.1111/acel.13217 (PMC7576262; doi:10.1111/acel.13217)

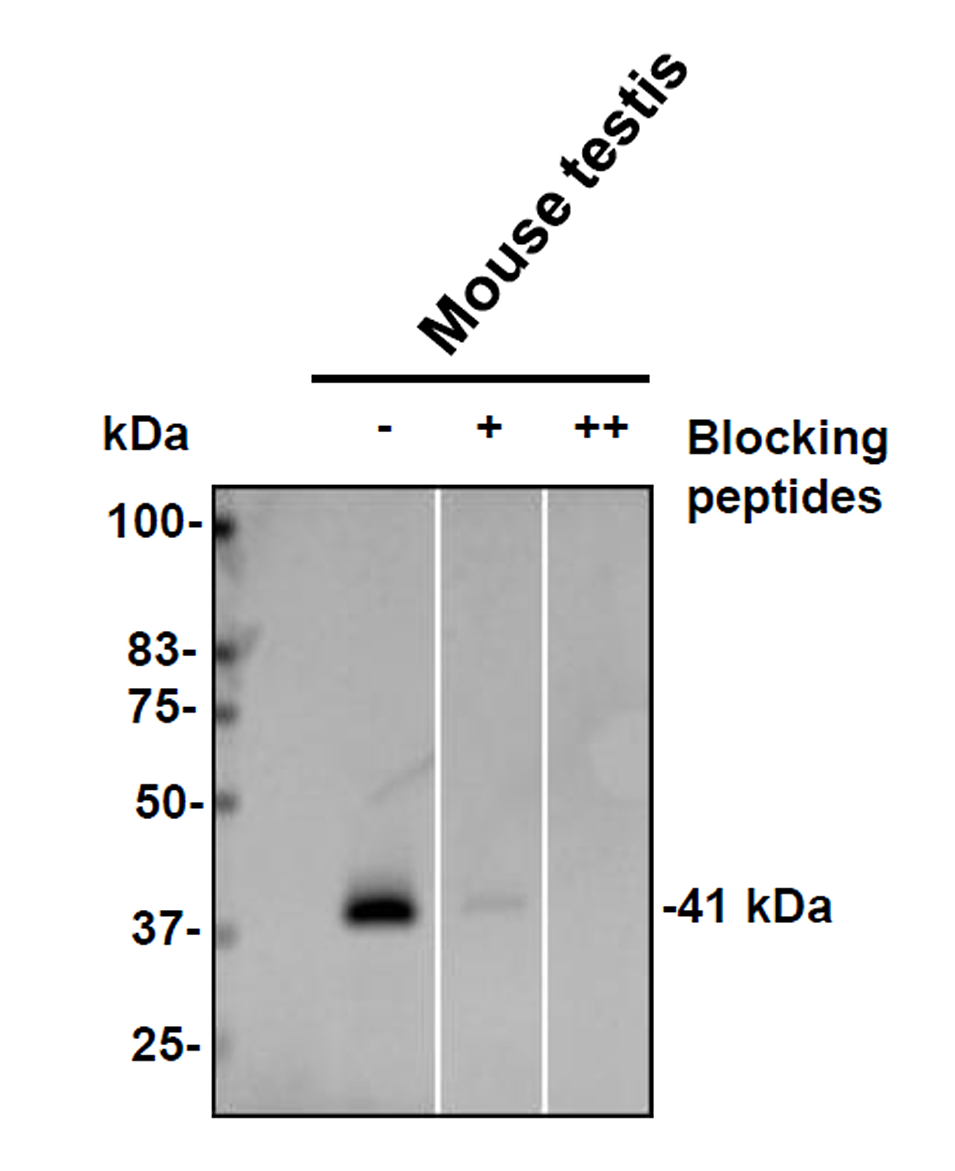

Supplement: Supplementary file 1 — Fig S1 [file ACEL-19-e13217-s001.tif]

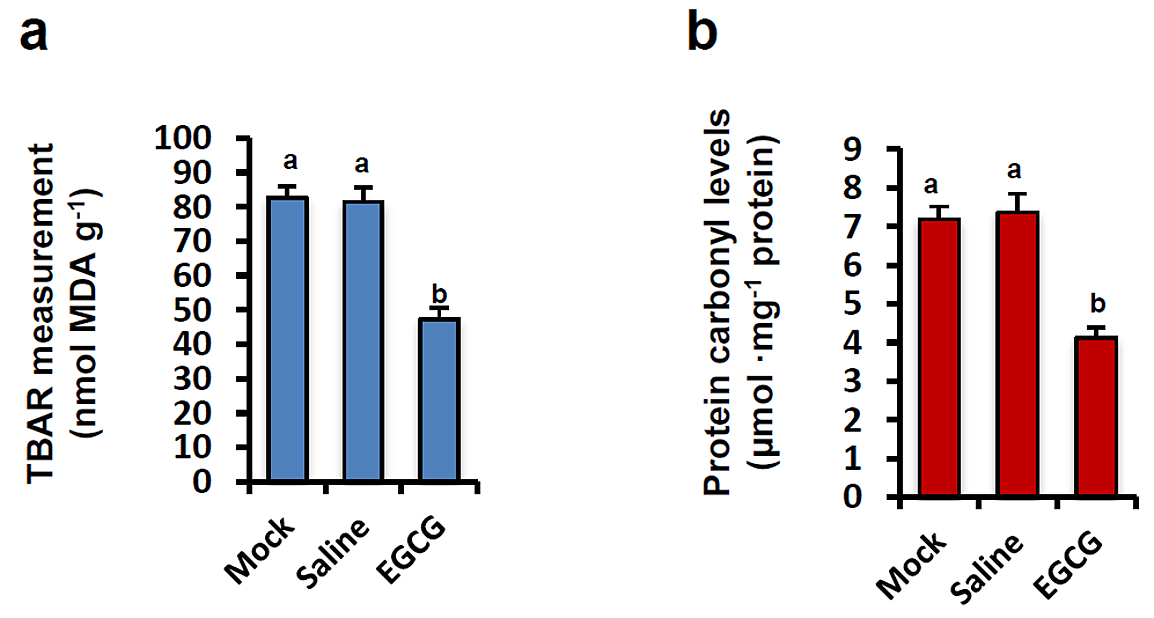

Supplement: Supplementary file 2 — Fig S2 [file ACEL-19-e13217-s002.tif]
