# Supplementary material for: Testicular Lmcd1 regulates phagocytosis by Sertoli cells through modulation of NFAT1/Txlna signaling pathway
Source: Aging Cell. 2020 Aug 9;19(10):e13217. doi: 10.1111/acel.13217 (PMC7576262; doi:10.1111/acel.13217)
Supplement: Supplementary file 3 — Table S1 [file ACEL-19-e13217-s003.doc]

**Supplementary Table 1**Assessment of male fertility and epididymal parameters after *in vivo* siRNA assay.

| **Experimental groups** | | **Reproductive capacity** | | | | | **Characteristics of epididymal sperms** | | | |  |
| --- | --- | --- | --- | --- | --- | --- | --- | --- | --- | --- | --- |
|  |  | **Pregnancies/**  **females mated** | | **Litter size** | **Number of males mated** | | **Number of sperm**  **(10^6^/epididymis)** | | **Progressive motility (%)** | |  |
| **Naive** | | 42/48 (87.5%) ^a^ | | 9.1 ± 2.6 ^a^ | 10 | | 34.8 ± 2.3 ^a^ | | 41.7 ± 4.6 ^a^ | |  |
| **Ctrl siRNA** | | 40/45 (88.9%) ^a^ | | 8.7 ± 1.9 ^a^ | 10 | | 32.3 ± 1.7 ^a^ | | 40.6 ± 2.2 ^a^ | |  |
|  | **Stealth siRNA** | | 7/38 (18.4%) ^b^ | 4.2 ± 1.3^b^ | | 10 | | 20.2 ± 4.8 ^b^ | | 19.4 ± 3.7 ^b^ | |

Different superscript letters denote groups that are statistically different in the same category (*P*< 0.05).
