# Supplementary material for: Testicular Lmcd1 regulates phagocytosis by Sertoli cells through modulation of NFAT1/Txlna signaling pathway
Source: Aging Cell. 2020 Aug 9;19(10):e13217. doi: 10.1111/acel.13217 (PMC7576262; doi:10.1111/acel.13217)
Supplement: Supplementary file 4 — Table S2 [file ACEL-19-e13217-s004.doc]

**Supplementary Table 2** Sources of antibodies and the working dilutions that were used for immunoblotting, immunostaining or immunoprecipitation in the current study

| Antibody | Vendor | | Catalog no. | Dilutions |
| --- | --- | --- | --- | --- |
| Rabbit anti-LMCD1 | | Novus (Shanghai, China) | NBP1-85986 | 1:1000 (IB)  1:300 (IHC) |
| Rabbit anti-CK18 | | Santa Cruz Biotechnology Inc. (Santa Cruz, CA) | sc-28264 | 1:1000 (IB) |
| Rabbit anti-TUBULIN | | Santa Cruz Biotechnology Inc. (Santa Cruz, CA) | sc-9104 | 1:2000 (IB) |
| Rabbit anti-TNFR1 | | Cell Signaling (Shanghai, China) | #13377 | 1:2000 (IB) |
| Rabbit anti-TXLNA | | Santa Cruz Biotechnology Inc. (Santa Cruz, CA) | sc-33762 | 1:1000 (IB)  1:200 (IHC) |
| Mouse anti-NFAT1 | | Thermo Fisher Scientific (Shanghai, China) | MA1-025 | 1:1000 (IB)  1:200 (IF) |
| Mouse anti-NFAT2 | | Thermo Fisher Scientific (Shanghai, China) | MA3-024 | 1:1000 (IB)  1:200 (IF) |
| Rabbit anti-p-NFAT1^Ser54^ | | Thermo Fisher Scientific (Shanghai, China) | PA1-27416 | 1:1000 (IB) |
| Mouse anti-DDK | | OriGene (Beijing, China) | TA50011-100 | 2μg/reaction tube (IP) |
| Rabbit anti-HA | | OriGene (Beijing, China) | TA150034 | 1:2000 (IB) |
| Goat anti-Mouse IgG-HRP secondary antibody | | Abcam (Hangzhou, China) | ab97023 | 1:10000 (IB) |
| Goat anti-Rabbit IgG-HRP secondary antibody | | Abcam (Hangzhou, China) | ab97200 | 1:10000 (IB) |
| VECTASTAIN® Elite® ABC HRP Kit | | VECTOR LABORATORIES, (Shenzhen, China) | PK-7200 | 1:500 (IHC) |
| Goat anti-Rabbit IgG-FITC secondary antibody | | Abcam (Hangzhou, China) | ab6785 | 1:1000 (IF) |
| Rabbit anti-pSTAT3^Y705^ | | Cell Signaling (Shanghai, China) | # 9131 | 2 μg/reaction tube (IP) |
| Rabbit anti-p16 | | Cell Signaling (Shanghai, China) | # 80772 | 1:1000 (IB) |
| Rabbit anti-p19 | | Cell Signaling (Shanghai, China) | #2156 | 1:1000 (IB) |
| Rabbit anti-PCNA | | Abcam (Hangzhou, China) | ab92552 | 1:2000 (IB) |
| Rabbit anti-CDK4 | | Cell Signaling (Shanghai, China) | #12790 | 1:2000 (IB) |
| Rabbit anti-γH2AX | | Abcam (Hangzhou, China) | ab2893 | 1:1000 (IB) |

IHC, immunohistochemistry/immunostaining; IB, Western blotting/immunoblotting; IP, immunoprecipitation; IF, immunofluorescence.
