# Supplementary material for: Testicular Lmcd1 regulates phagocytosis by Sertoli cells through modulation of NFAT1/Txlna signaling pathway
Source: Aging Cell. 2020 Aug 9;19(10):e13217. doi: 10.1111/acel.13217 (PMC7576262; doi:10.1111/acel.13217)
Supplement: Supplementary file 5 — Table S3 [file ACEL-19-e13217-s005.doc]

**Supplementary Table 3 Primers used for the RT-qPCR analysis**

| **Target gene** | **GenBank no. or references** | **Primer sequence (5'-3')** |
| --- | --- | --- |
| *Lmcd1* | NM_144799.2 | F: 5’-AAGAGGAGTGGCTTGTCTGC-3’  R: 5’-CCGATCATCGTCCAGGTCAG-3’ |
| *Gapdh* | Reference [1] | F: 5’-GGGTGAGGCCGGTGCTGAGT-3’  R: 5’-TGACCCGTTTGGCTCCACCCT-3’ |
| *Scarb1* | Reference [2] | F: 5’-ATGCCCCAGGTTCTTCACTAC-3’  R: 5’-CTGGTGACATCAGGGACTCAG-3’ |
| *Nhlrc2* | NM_025811.3 | F: 5’-TCACCATTGCTGTTCCCTGG-3’  R: 5’-AGGGTTAGGTCCTCCAATGC-3’ |
| *Mta2* | Reference [3] | F: 5’-TGGTTAGACGGATTGAGGAG-3’  R: 5’-TCAAACTCCCGAGCATTACT-3’ |
| *Txlna* | Reference [4] | F: 5’-GTGGCATTCGGGAGAAAG-3’  R: 5’-AGAAATCCCCAAATCTGACG-3’ |
| *Mertk* | NM_008587.2 | F: 5’-ACGTTGGTGGATACGTGCAT-3’  R: 5’-CTCTTCCCACTTCTCGGCAG-3’ |
| *Elmo1* | NM_198093.3 | F: 5’-GGGTCTCTGTTCACATGGCT-3’  R: 5’-TAGAGTCGTGCCCCAGTAGG-3’ |
| *Dnm2* | NM_001253893.1 | F: 5’-GAGGGCCATACCCAATCAGG-3’  R: 5’-GACTCAGCTGTCAGCACGAA-3’ |
| P1 | NC_000070.6 | F: 5’-ATGTTAGACATTTCAATAAGGTGTG-3’  R: 5’-TGCTCATATAAGCCTAGCTGA-3’ |
| P2 | NC_000070.6 | F: 5’-ACACCCACCGCCACTCATGC-3’  R: 5’-TGTCCTTCTCAGGACTTCTGC-3’ |
| P3 | NC_000070.6 | F: 5’-ACAGCTGAGTAAGGGGTAGGAA-3’  R: 5’-CAGCTGAATTAGGGAACTACT-3’ |
| P4 | NC_000070.6 | F: 5’-AAGCAGCTGAAGGTGGCAAC-3’  R: 5’-AGCACGCTGGAAGGTCCCT-3’ |
| ChIP for *Lmcd1* | NC_000072.6 | F: 5’-ACTCAGAACTAGATCTCCAGGTCT-3’  R: 5’-TACCTTTGACTACAACCATCAAG-3’ |

1. Zhang LL, Ma J, Yang B, Zhao J, Yan BY, Zhang YQ, Li W. Interference with lactate metabolism by mmu-miR-320-3p via negatively regulating GLUT3 signaling in mouse Sertoli cells. Cell Death Dis. 2018; 9: 964.

2. Akita N, Tsujita M, Yokota T, Gonzalez FJ, Ohte N, Kimura G, Yokoyama S. High density lipoprotein turnover is dependent on peroxisome proliferator-activated receptor alpha in mice. J Atheroscler Thromb. 2010; 17: 1149-1159.

3. Zhang S, Li W, Zhu C, Wang X, Li Z, Zhang J, Zhao J, Hu J, Li T, Zhang Y. Sertoli cell-specific expression of metastasis-associated protein 2 (MTA2) is required for transcriptional regulation of the follicle-stimulating hormone receptor (FSHR) gene during spermatogenesis. J Biol Chem. 2012; 287: 40471-40483.

4. Dong YS, Hou WG, Li Y, Liu DB, Hao GZ, Zhang HF, Li JC, Zhao J, Zhang S, Liang GB, Li W. Unexpected requirement for a binding partner of the syntaxin family in phagocytosis by murine testicular Sertoli cells. Cell Death Differ. 2016; 23: 787-800.
