# Supplementary material for: Testicular Lmcd1 regulates phagocytosis by Sertoli cells through modulation of NFAT1/Txlna signaling pathway
Source: Aging Cell. 2020 Aug 9;19(10):e13217. doi: 10.1111/acel.13217 (PMC7576262; doi:10.1111/acel.13217)
Supplement: Supplementary file 6 — Supplementary Material [file ACEL-19-e13217-s006.doc]

**Supporting Information**

**SI Materials and Methods**

**Histological examination**

Localization of different target proteins in testis was revealed by immunohistochemistry (Li et al., 2015). After routine deparaffin and rehydration, sections were treated with boiling antigen-unmasking solution (Vector Labs, Shanghai, China) for 0.5 h at 95°C. After being cooled slowly to room temperature, sections were incubated with incubated with different primary antibodies (**Supplementary Table 2**) in PBS at 4°C overnight, followed by sequential incubation with biotinylated second antibody and streptavidin peroxidase complex provided by VECTASTAIN® Elite® ABC HRP Kit (Vector Labs).

To reveal the subcellular localization of NFAT2 and NFAT1 in SCs, cells were fixed with 4% paraformaldehyde at 25°C for 20 min. After treatment with the blocking solution (10% donkey serum, 0.5% BSA and 0.3% Triton X-100 in PBS) for 0.5 h, cells were incubated with different primary antibodies (**Supplementary Table 2**) in blocking solution at 4°C overnight. The immunoreactions were then developed by incubation with goat anti-rabbit FITC 488-conjugated IgG (Sigma-Aldrich), followed by observation under an inverted microscope (Axio Imager M1 microscope, Zeiss, Germany).

Hematoxylin-eosin (HE), TUNEL and Oil Red O staining in testicular sections were carried out according to our previous work (Dong et al., 2016).

**Measurement of testicular oxidative stress**

The oxidative stress status in different testicular samples was determined by measuring lipid peroxidation and protein carbonyl levels according to our previous report (Ding et al., 2015). Final absorbance was measured in triplicate at 535 and 370 nm respectively, on a microplate reader (Bio-Rad, Shanghai, China).

**Lipid accumulation and cellular senescence determination**

15P-1^Lmcd1 sh^ and Ctrl cells were incubated with apoptotic GCs for 3 days. Subsequent SCs senescence was measured by determining the senescence-associated-beta-galactosidase activity (SA-ß-GAL) activity using a Cellular Senescence Activity Assay kit (Enzo Life Sciences, Shanghai, China), as per the manufacturer’s instructions. To detect the cellular neutral lipid contents in the above-mentioned 15P-1^Lmcd1 sh^ and Ctrl cells, cells were harvested upon completion of co-incubation with apoptotic GCs. Cells were then stained by being resuspended in 500 μl of BODIPY 493/503 at 0.5 μg/ml in PBS at 20°C. 15 minutes later, cells were harvested and subjected to assessment of the geometric mean fluorescence intensity (MFI) of BODIPY on a FLx800 Fluorescence Reader (BioTek, Beijing, China).

**RT-qPCR**

Total RNA was prepared using RNeasy Mini kit (Qiagen, Shanghai, China). After a routine DNase treatment, RNA samples were subjected to synthesis of first-strand cDNA using SuperScript III (Thermo Fisher Scientific). Subsequent PCR was performed according to Promega's reverse transcription system protocol. The primers used were listed in **Supplementary Table 3**. PCR products were quantified by SYBR green intercalation, and amplification of *Gapdh* was used to obtain the ^△△^Ct values for the calculation of fold changes.

**Immunoblotting**

Total protein was isolated using ReadyPrep™ Kit (Bio-Rad, Shanghai, China). The nuclear and cytoplasmic protein was purified using NE-PER™ Nuclear and Cytoplasmic Extraction kit (Thermo Fisher Scientific). After protein quantification by NanoDrop, ~30 μg of protein samples were separated on SDS-PAGE and transferred to PVDF membranes (Sigma-Aldrich). Membranes were then hybridized with primary antibodies (**Supplementary Table 2**) at 4°C overnight, followed by signal development using ONE-HOUR Western™ System (GenScript, Nanjing, China).

**Luciferase reporter assay**

The genomic fragments of mouse *Txlna* promoter (~1.9 kb upstream of the translation start site) and a ~2.1 kb mouse *Lmcd1* promoter were amplified from genomic DNA of mouse testis and were subcloned into pGL4-Basic vector (Promega, Madison, WI, USA) using CloneJET PCR Cloning Kit (Thermo Fisher Scientific). For reporter assay, 0.5 μg reporter plasmid and pRL-TK Renilla reporter plasmid were co-transfected into 15P-1 cells with different plasmids as indicated. 48 h later, cells were incubated with apoptotic GCs for 6 h, followed by measurement of luciferase activities using a dual luciferase reporter assay kit (Promega).

**Non-radioactive electrophoretic mobility shift assay (EMSA)**

15P-1 cells were transfected with Stat3 Flag pRc/CMV which renders the STAT3 molecule constitutively active (Bromberg et al., 1999). 48 h after transfection, 15P-1 cells were treated with 1 mM of diamide for another 6 h. Subsequently, cells were harvested and nuclear protein extracts were prepared using the Nuclear Extraction Kit (Abcam) following the vendor’s protocol. To test direct binding of STAT3 to a specific site on the *Lmcd1* promoter *in vitro*, 1 μg of STAT3-enriched nuclear extracts was incubated with 30 mM of custom DIG-labeled probes corresponding to the putative STAT3 binding motifs. Upon completion of gel electrophoresis and nylon membrane transfer, protein-bound DIG-labeled probes were immunologically detected with anti-DIG–alkaline phosphatase conjugate and CSPD chemiluminescent substrate (Roche), as per the manufacturer’s instructions. The oligonucleotide probe used was AACTAATGTGCTCTGAGCTTCCCTTATATGCTTGAT. Competitive inhibition consisted of incubation with a 50-fold molar excess of unlabeled/cold probe.

**Co-Immunoprecipitation (Co-IP) and chromatin immunoprecipitation (ChIP)**

15P-1 cells with different transfections were stimulated with apoptotic GCs for 6 h. Cells were then harvested and subjected to Co-IP and ChIP assay, as described in detail in our previous work (Zhang et al., 2012).

**Statistical analysis**

Quantitative data that were expressed as mean ± S.D. were analyzed for statistical difference using *Student's t*-test or one-way analysis of variance wherever appropriate. Statistical analysis was performed with the aid of SPSS 19.0 software and *P*<0.05 was considered statistically significant.

Bromberg, J. F., Wrzeszczynska, M. H., Devgan, G., Zhao, Y., Pestell, R. G., Albanese, C. (1999). Stat3 as an oncogene. *Cell, 98*(3), 295-303. doi:S0092-8674(00)81959-5 [pii]

10.1016/s0092-8674(00)81959-5

Ding, J., Wang, H., Wu, Z. B., Zhao, J., Zhang, S., & Li, W. (2015). Protection of murine spermatogenesis against ionizing radiation-induced testicular injury by a green tea polyphenol. *Biol Reprod, 92*(1), 6. doi:biolreprod.114.122333 [pii]

10.1095/biolreprod.114.122333

Dong, Y. S., Hou, W. G., Li, Y., Liu, D. B., Hao, G. Z., Zhang, H. F. (2016). Unexpected requirement for a binding partner of the syntaxin family in phagocytosis by murine testicular Sertoli cells. *Cell Death Differ, 23*(5), 787-800. doi:cdd2015139 [pii]

10.1038/cdd.2015.139

Li, W., Fu, J., Zhang, S., Zhao, J., Xie, N., & Cai, G. (2015). The proteasome inhibitor bortezomib induces testicular toxicity by upregulation of oxidative stress, AMP-activated protein kinase (AMPK) activation and deregulation of germ cell development in adult murine testis. *Toxicol Appl Pharmacol, 285*(2), 98-109. doi:S0041-008X(15)00121-0 [pii]

10.1016/j.taap.2015.04.001

Zhang, S., Li, W., Zhu, C., Wang, X., Li, Z., Zhang, J. (2012). Sertoli cell-specific expression of metastasis-associated protein 2 (MTA2) is required for transcriptional regulation of the follicle-stimulating hormone receptor (FSHR) gene during spermatogenesis. *J Biol Chem, 287*(48), 40471-40483. doi:M112.383802 [pii]

10.1074/jbc.M112.383802
