# Supplementary material for: Testicular Lmcd1 regulates phagocytosis by Sertoli cells through modulation of NFAT1/Txlna signaling pathway
Source: Aging Cell. 2020 Aug 9;19(10):e13217. doi: 10.1111/acel.13217 (PMC7576262; doi:10.1111/acel.13217)
Supplement: Supplementary file 7 — Fig S1‐S2‐Cap [file ACEL-19-e13217-s007.docx]

**Figure S1** Immunoblotting analysis in adult mouse testis demonstrated a single band of LMCD1 protein in the whole blot, whereas preabsorption with blocking peptides effectively abolished the positive staining, confirming the specificity of the antibody.

**Figure S2** 540-day-old mice received i.p. injections with EGCG (50 mg/kg) for consecutive 3 days, followed by a 4-day break. Mice received a total 4 cycles of EGCG treatment. After EGCG treatment, testicular oxidative stress were determined in tissue homogenates by measuring lipid peroxidation (a) and protein carbonyl levels (b), as described by our previous work (Ding et al., 2015). Statistically significant differences from mock controls, as determined by *Student t*-test, are indicated using different superscript letters (*P*<0.05).
